# Case management of patients with Type 2 diabetes mellitus: a cross-sectional survey in Chongqing, China

**DOI:** 10.1186/s12913-017-2039-0

**Published:** 2017-02-11

**Authors:** Miao He, Jiaqi Gao, Weiwei Liu, Xiaojun Tang, Shenglan Tang, Qian Long

**Affiliations:** 1 School of Public Health and Management, Collaborative Innovation Center of Social Risks Governance in Health, Research Center for Medicine and Social Development, Chongqing Medical University, No.1 Yixueyuan Road, Chongqing, Yuzhong district 400016 People’s Republic of China; 2 Duke Global Health Institute, Duke University, Durham, NC USA; 3 Global Health Research Center, Duke Kunshan University, Kunshan, China

**Keywords:** Type 2 diabetes mellitus, Chronic disease case management, Compliance, China

## Abstract

**Background:**

Type 2 diabetes mellitus has been identified as one of the priority diseases and included in the essential public health service package in China. This study investigated the frequency of follow-up visits and contents of care for case management of patients with Type 2 diabetes in Chongqing located in the western China, in terms of the regional practice guideline; and analyzed factors associated with the use of care.

**Methods:**

A cross-sectional survey was conducted with patients diagnosed with Type 2 diabetes in two areas in Chongqing. Total 502 participants (out of 664 people eligible) completed the interview. The outcome measures included at least four follow-up visits in a year, annual HbA1c test, blood lipid test and diabetic screening for nephropathy and eyes. Logistic regression analysis was applied to examine the association between participants’ demographic and socio-economic characteristics and outcome measures.

**Results:**

Over the one-year study period, 65% of participants had four or more follow-up visits. In light of the recommended tests, the proportions of having HbA1c test, blood lipid test and screening for nephropathy and eyes annually were 8, 54, 45 and 44%, respectively. After adjusting for study sites, age, sex, education, type of residence, level of income, the patients who were covered by Urban Employee Basic Medical Insurance, were enrolled in the targeted disease reimbursement program, and lived with diabetes more than five years were more likely to have regular follow-up visits and the recommended tests.

**Conclusions:**

Case management for patients with Type 2 diabetes mellitus was not effectively implemented in terms of frequency of follow-up visits and recommended tests over one-year period, as indicated in the regional practice guideline.

## Background

Diabetes mellitus (DM) is one of the top ten causes of disability in the world and undermines productivity and human development [1]. In China, it is estimated that the prevalence of diabetes is 9.4% in 2016 and approximately 224,700 people die from diabetes [2]. It is therefore particularly important that the public health system ensures the delivery of effective healthcare services to those with the disease.

China’s National Plan for Non-Communicable Diseases (NCDs) Prevention and Treatment (2012–2015) adopts primary healthcare approach highlighting the importance of early diagnosis and early treatment of NCDs [3]. Type 2 DM has been identified as one of the priority diseases in China. According to the national guideline for implementation of the basic public health service package, case management should be provided with patients with Type 2 DM once a quarter aiming to monitor the disease progression, guide the treatment and promote healthy lifestyle [4]. The recommended services for case management included blood glucose test and other relevant examinations, routine physical check-up, health education and nutrition guidance.

In China, there are three basic health insurance schemes covering over 95% of the population. They are Urban Employee Basic Medical Insurance (UEBMI), Urban Residence Basic Medical Insurance (URBMI) and New Rural Cooperative Medical Scheme (NCMS) [5, 6]. UEBMI has relatively comprehensive coverage of health services. URBMI and NCMS largely cover inpatient care, and in the recent years they also provide outpatient coverage, albeit the benefit package being modest [7].

Chongqing is one of the largest cities located in the western China, with a total population of over 30 million. The prevalence of diabetes in Chongqing was 10.7% in 2014 [8]. In 2010, the two schemes, URBMI and NCMS were integrated in one scheme, named as Urban–rural Residence Basic Medical Insurance (URRBMI) in Chongqing. This scheme and UEBMI have a targeted disease reimbursement program to provide a higher level of reimbursement for outpatient care for 20 priority disease, including DM [5]. In line with the national plan for NCDs management and treatment, the provincial government has issued a practice guideline targeting on management of elder people and patients with Type 2 DM and hypertension, which has introduced a set of indicators for monitoring and evaluation of NCDs management [9]. Regarding the diabetic case management, the guideline recommended one face-to-face visit every quarter and a comprehensive check-up and in a year (including routine blood and urine tests, electrocardiogram, liver and kidney function examinations, HbA1c test and diabetic eyes screening).

Many previous studies in China focused on the treatment of Type 2 DM [10, 11] but few studies examined case management for Type 2 DM patients. This study investigated the frequency of follow-up visits and contents of care for case management of patients with Type 2 DM in Chongqing, in terms of regional practice guideline; and analyzed factors associated with the use of care.

## Methods

### Study sites

The cross-sectional patient survey was carried out in two areas where the NCDs management and registry systems have been established. The two selected areas represented, respectively, less developed area (DJ County) and developed one (YB District) in Chongqing. Table 1 presents the basic characteristics of the study sites. One township and one community were selected in each area which was near to and far from county/district hospital, and the local health authorities would like to participate in the study.

**Table 1:** Characteristics of study sites, 2013

Items	DJ county	YB district
Administrative divisions	25 townships 1 community	11 townships 18 communities
Selected sites	One township (GF): around 25 km from the county hospital	One township (LX): around 16 km from the district hospital
	One community (GX): nearest to the county hospital	One community (SFQ): nearest to the district hospital
Population	Around 970,900	Around1,136,900
Urban	226,100	712,900
Rural	744,800	424,000
Local gross domestic product per capita (US$)^a^	4612.90	11145.16

^a^Gross domestic product per capita in Chongqing was 6903.23 US dollars in 2013

### Participants and data collection

Eligible participants were men and women who have been diagnosed with Type 2 DM before 2013 and were registered in the chronic disease management system at the health centers. Pregnant women with gestational DM were excluded. The total number of eligible participants was 664. The survey was carried out between June to September 2014. Trained university students and teachers conducted the interview using a structured questionnaire developed by the research team, which was finalized after the pilot test. The questions included demographic and socioeconomic characteristics of participants, years of patients living with DM, frequency of having follow-up visits in a year, contents of care (referred to the local practice guideline) and expenditures of health services used. Staffs working in the health centers contacted and invited all eligible participants to come to a place where was convenient for patients. The interviews were conducted using local dialect in a private room. Total 502 participants (out of 664) completed the interview.

### Data analysis

In the study, outcome measures referred to the recommendations of the regional practice guideline for the management of Type 2 DM. They were at least four follow-up visits in a year, HbA1c test, blood lipid test, diabetic eye and nephropathy screening once a year, respectively. Participants who had less than four follow-up visits or didn’t have any of test mentioned above were considered as sub-standard management. Explanatory variables included participants’ age (≤59,60-69,≥70), gender (male and female), type of residence (urban, rural), education (illiterate, primary school, middle school or higher), level of income (low, middle and high), type of health insurance (UEBMI or URRBMI), the enrollment status of the targeted disease reimbursement program (yes, no) and years of living with Type 2 DM (≤5, >5). Income category was grouped, using annual per capita income. The reported annual household income (which represented total annual consumption and savings in a calendar year prior to the survey) was divided by the number of family members and grouped into three income categories (DJ County and YB District respectively), each containing a third of patients. The targeted disease reimbursement program in health insurance schemes provided a higher level of reimbursement for DM outpatient and/or inpatient care for those who were diagnosed with DM and requested to enroll in this program. The more details of the targeted disease reimbursement program were reported elsewhere [5].

The completed questionnaires were cross-checked, and any logical mistakes were corrected. The data were double-entered into the database (EpiData version 3.1). Cross tabulation was used to compare follow-up management of DM in the two study sites. The multi-collinearity diagnosis was performed showing no evidence of multi-collinearity among variables. The multi-variable logistical regression (stepwise selection) was used to examine the associations between all explanatory variables and outcome measures. Data without statistically significant were not shown. Odds Ratios (OR) and their 95% confidence intervals (95% CI) were calculated. The SAS 9.1.3 was used for statistical analyses.

This study was approved by the Research Ethics Committee of Chongqing Medical University. Written consents of participants were obtained for data collection.

## Results

### Demographic and socioeconomic characteristics

Of 502 participants, six participants did not complete the section of content of care for the case management in the questionnaire. Thus, total 496 participants were included in this analysis. Table 2 presents the characteristics of participants. In the both study sites, a vast majority of participants were over 60 years old and there were more women than men. All participants had health insurance coverage: three-fourth of them covered by the URRBMI, while less than one-third of all participants were enrolled in the targeted disease reimbursement program. Around half of the participants lived with Type 2 DM less than five years. Compared to YB, more participants lived in rural areas were illiterate in DJ.

**Table 2:** Demographic and socioeconomic characteristics of patients with Type 2 DM in Chongqing, 2013

Variables		DJ county N(%)	YB district N(%)	Total N(%)	*χ* ^2^	*P*
Type of residence	Rural	146 (65.77)	51 (18.61)	197 (39.72)	113.88	<0.01
	Urban	76 (34.23)	223 (81.39)	299 (60.28)		
Age (years)	≤59	52 (23.42)	65 (23.72)	117 (23.59)	1.09	0.58
	60 ~ 69	91 (40.99)	123 (44.89)	214 (43.15)		
	≥70	79 (35.59)	86 (31.39)	165 (33.27)		
Sex	Male	95 (42.79)	105 (38.32)	200 (40.32)	1.02	0.31
	Female	127 (57.21)	169 (61.68)	296 (59.68)		
Education ^a^	Middle school or higher	82 (37.79)	95 (34.67)	177 (36.05)	12.66	<0.01
	Primary school	64 (29.49)	120 (43.80)	184 (37.47)		
	Illiterate	71 (32.72)	59 (21.53)	130 (26.48)		
Income category ^b^	High	73 (33.03)	90 (33.46)	172 (35.10)	1.81	0.40
	Middle	75 (33.94)	104 (38.66)	156 (31.84)		
	Low	73 (33.03)	75 (27.88)	162 (33.06)		
Type of health insurance ^c^	UEBMI	54 (24.43)	73 (26.64)	127 (25.66)	0.31	0.58
	URRBMI	167 (75.57)	201 (73.36)	368 (74.34)		
Involved in the targeted disease reimbursement program ^d^	Yes	68 (31.05)	85 (31.02)	153 (31.03)	0.00	0.99
	No	151 (68.95)	189 (68.98)	340 (68.97)		
Years living with Type 2 DM	≤5	116 (52.25)	155 (56.57)	271 (54.64)	0.92	0.34
	>5	106 (47.75)	119 (43.43)	225 (45.36)		

^a^ Data was missing for five patients

^b^ Data was missing for six patients. In DJ, high income level: >US$1900.6; middle income level: US$542.2–1900.6; low income level: <US$542.2; in YB, high income level: >US$2580.6; middle income level: US$1548.4–2580.6; low income level: <US$1548.4

^c^ Data was missing for one patients

^d^ Data was missing for three patients

### The case management of Type 2 DM

Around two-third of participants had four or more follow-up visits (Mean ± Standard deviation, 8 ± 5) in a year as recommended. The proportion of having at least one HbA1c test annually was only 8.06%. Around half of participants had blood lipid test and diabetic screening for nephropathy and eyes at least once a year (Table 3). Compared to YB, the proportion of having four or more follow-up visits was slightly higher in DJ, while the proportions of having recommended tests were slightly lower. The differences between the two study sites were not statistically significant (Table 3).

**Table 3:** The case management of patients with Type 2 DM in two study sites, 2013

	Total standard rate (%)	Areas	Standard^a^ N	Sub-standard N	Standard rate (%)	*χ* ^2^	*P*
Follow-up once a quarter ^b^	64.99	DJ County	146	71	67.28	0.92	0.34
		YB District	164	96	63.08		
HbA1c test once a year	8.06	DJ County	13	209	5.86	2.64	0.10
		YB District	27	247	9.85		
Blood lipid test once a year	54.23	DJ County	114	108	51.35	1.35	0.25
		YB District	155	119	56.57		
Screening for nephropathy once a year	44.56	DJ County	92	130	41.44	1.58	0.21
		YB District	129	145	47.08		
Screening for eyes once a year	43.55	DJ County	88	134	39.64	2.50	0.11
		YB District	128	146	46.72		

^a^ According to the regional practice guideline for the case management of patients with Type 2 DM, standard management was defined as patients having at least four follow-up visits in a year and having the recommended tests once a year

^b^ Data was missing for 19 patients

### Factors associated with non-adherence of the regional practice guidelines

#### Follow-up visits

In the univariate analysis, participants who were not enrolled in the targeted disease reimbursement program and lived with DM less than five years were more likely to have less than four follow-up visits in a year (Table 4). Similar results were found in the multivariate analysis. After adjusting for all explanatory variables, patients who were not enrolled in the targeted disease reimbursement program were three times less likely to have four follow-up visits in a year than their counterparts (OR 2.98, 95%CI 1.85–4.81) (Table 4). Patients lived with DM less than five years were 1.6 times less likely to have four follow-up visits in a year than patients lived with DM more than five years (OR 1.60, 95%CI 1.07–2.39) (Table 4).

**Table 4:** Factors affecting follow-up visits in a year, 2013

Variables	Type	Less four follow-up visits N(%)	Four or more follow-up visits N(%)	Unadjusted OR /95%CI	*P*	Adjusted OR /95%CI^c^	*P*
Involved in the targeted disease reimbursement program ^a^	Yes	27 (18.37)	120 (81.63)	1.00	—	1.00	—
	No	139 (42.51)	188 (57.49)	3.29 (2.05 ~ 5.27)	<0.01	2.98 (1.85 ~ 4.81)	<0.01
Years living with Type 2 DM ^b^	>5	60 (27.40)	159 (72.60)	1.00	—	1.00	—
	≤5	107 (41.47)	151 (58.53)	1.88 (1.28 ~ 2.76)	<0.01	1.60 (1.07 ~ 2.39)	0.02

^a^ Data was missing for 22 patients

^b^ Data was missing for 19 patients

^c^ Adjusted for study sites, age, sex, education, type of residence, level of income, type of health insurance, status of enrollment in the targeted disease reimbursement program and years of living with Type 2 DM. Data without statistically significant were not shown

#### Annual HbA1c test

In the univariate analysis, rural residence, patients with no education or illiterate, patients from low income group or having URRBMI and non-enrolled in the targeted disease reimbursement program were less likely to have annual HbA1c test (Table 5). After adjusting for all explanatory variables, only type of health insurance was significantly associated with having HbA1c test. Patients covered by URRBMI were five times less likely to have HbA1c test than patients covered by UEBMI (OR 5.40, 95% CI 2.73–10.69) (Table 5).

**Table 5:** Factors affecting Annual HbA1c test in a year, 2013

Variables	Type	Non-annual HbA1c test N(%)	Annual HbA1c test N(%)	Unadjusted OR/95%CI	*P*	Adjusted OR/95%CI^e^	*P*
Type of residence	Urban	268 (89.63)	31 (10.37)	1.00	—	—	—
	Rural	188 (95.43)	9 (4.57)	2.42 (1.12 ~ 5.19)	0.02	NA	NA
Education ^a^	Middle school or higher	154 (87.01)	23 (12.99)	1.00	—	—	—
	Primary school	175 (95.11)	9 (4.89)	2.90 (1.30 ~ 6.47)	0.01	NA	NA
	Illiterate	122 (93.85)	8 (6.15)	2.28 (0.98 ~ 5.27)	>0.05	NA	NA
Income category ^b^	High	140 (85.89)	23 (14.11)	1.00	—	—	—
	Middle	171 (95.53)	8 (4.47)	3.51 (1.52 ~ 8.09)	<0.01	NA	NA
	Low	140 (94.59)	8 (5.41)	2.88 (1.24 ~ 6.65)	0.01	NA	NA
Type of health insurance ^c^	UEBMI	103 (81.10)	24 (18.90)	1.00	—	—	—
	URRBMI	352 (95.65)	16 (4.35)	5.13 (2.62 ~ 10.01)	<0.01	5.40 (2.73 ~ 10.69)	<0.01
Involved in the targeted disease reimbursement program ^d^	Yes	135 (88.24)	18 (11.76)	1.00	—	—	—
	No	318 (93.53)	22 (6.47)	1.93 (1.00 ~ 3.71)	<0.05	NA	NA

^a^ Data was missing for five patients

^b^ Data was missing for six patients

^c^ Data was missing for one patients

^d^ Data was missing for three patients

^e^ Adjusted for study sites, age, sex, education, type of residence, level of income, type of health insurance, status of enrollment in the targeted disease reimbursement program and years of living with Type 2 DM. Data without statistically significant were not shown

#### Annual blood lipid test

The patients who were covered by URRBMI and lived with DM less than five years were less likely to have blood lipid test in a year (Table 6). After adjusting for all explanatory variables, patients with URRBMI coverage were almost two times less likely to have blood lipid test than those with UEBMI coverage (OR 1.85, 95%CI 1.21–2.81); while the years of living with DM was not statistically significant in relation to have this test (Table 6).

**Table 6:** Factors affecting annual blood lipid test in a year, 2013

Variables	Type	Non-annual blood lipid test N(%)	Annual blood lipid test N(%)	Unadjusted OR/95%CI	*P*	Adjusted OR/95%CI^b^	*P*
Type of health insurance ^a^	UEBMI	44 (34.65)	83 (65.35)	1.00	—	—	—
	URRBMI	182 (49.46)	186 (50.54)	1.85 (1.21 ~ 2.81)	<0.01	1.85 (1.21 ~ 2.81)	<0.01
Years living with Type 2 DM	>5	92 (40.89)	133 (59.11)	1.00	—	—	—
	≤5	135 (49.82)	136 (50.18)	1.44 (1.00 ~ 2.05)	<0.05	NA	NA

^a^ Data was missing for one patients

^b^ Adjusted for study sites, age, sex, education, type of residence, level of income, type of health insurance, status of enrollment in the targeted disease reimbursement program and years of living with Type 2 DM. Data without statistically significant were not shown

### Annual diabetic nephropathy screening

Those patients who were rural residence, illiterate, were covered by URRBMI, were not enrolled in the targeted disease reimbursement program and lived with DM less than five years, were less likely to have diabetic nephropathy screening (Table 7). After adjusting for all explanatory variables, only rural residence (OR 1.74, 95%CI 1.19–2.55) and the patients non-enrolled in the targeted disease reimbursement program (OR 1.55, 95%CI 1.05–2.31) were statistically significantly associated with not having this test (Table 7).

**Table 7:** Factors affecting Annual screening for nephropathy in a year, 2013

Variables	Type	Non-annual screening for nephropathy N(%)	Annual screening for nephropathy N(%)	Unadjusted OR/95%CI	*P*	Adjusted OR/95%CI^d^	*P*
Type of residence	Urban	150 (50.17)	149 (49.83)	1.00	—	—	—
	Rural	125 (63.45)	72 (36.55)	1.73 (1.19 ~ 2.49)	<0.01	1.74 (1.19 ~ 2.55)	<0.01
Education ^a^	Middle school or higher	88 (49.72)	89 (50.28)	1.00	—	—	—
	Primary school	102 (55.43)	82 (44.57)	1.26 (0.83 ~ 1.90)	0.28	NA	NA
	Illiterate	85 (65.38)	45 (34.62)	1.91 (1.20 ~ 3.05)	0.01	NA	NA
Type of health insurance ^b^	UEBMI	55 (43.31)	72 (56.69)	1.00	—	—	—
	URRBMI	219 (59.51)	149 (40.49)	1.92 (1.28 ~ 2.89)	<0.01	NA	NA
Involved in the targeted disease reimbursement program ^c^	Yes	71 (46.41)	82 (53.59)	1.00	—	—	—
	No	204 (60.00)	136 (40.00)	1.73 (1.18 ~ 2.55)	0.01	1.55 (1.05 ~ 2.31)	0.03
Years living with Type 2 DM	>5	113 (50.22)	112 (49.78)	1.00	—	—	—
	≤5	162 (59.78)	109 (40.22)	1.47 (1.03 ~ 2.11)	0.03	NA	NA

^a^ Data was missing for five patients

^b^ Data was missing for six patients

^c^ Data was missing for three patients

^d^ Adjusted for study sites, age, sex, education, type of residence, level of income, type of health insurance, status of enrollment in the targeted disease reimbursement program and years of living with Type 2 DM. Data without statistically significant were not shown

#### Annual diabetic eyes screening

Likewise, the patients who were rural residence, illiterate, were covered by URRBMI and were not enrolled in the targeted disease reimbursement program were less likely to have diabetic eyes screening (Table 8). In multivariate analysis, patients covered by URRBMI were less likely to have this test than those covered by UEBMI (OR 2.07, 95%CI 1.37–3.12) (Table 8). There were no statistically significant association between other variables and having this test.

**Table 8:** Factors affecting annual diabetic eyes screening in a year, 2013

Variables	Type	Non-Annual diabetic eyes screening N(%)	Annual diabetic eyes screening N(%)	Unadjusted OR/95%CI^*^	*P*	Adjusted OR/95%CI^d^	*P*
Type of residence	Urban	154 (51.51)	145 (48.49)	1.00	—	—	—
	Rural	126 (63.96)	71 (36.04)	1.67 (1.16 ~ 2.42)	0.01	NA	NA
Education ^a^	Middle school or higher	90 (50.85)	87 (49.15)	1.00	—	—	—
	Primary school	105 (57.07)	79 (42.93)	1.29 (0.85 ~ 1.95)	0.24	NA	NA
	Illiterate	85 (65.38)	45 (34.62)	1.83 (1.15 ~ 2.91)	0.01	NA	NA
Type of health insurance ^b^	UEBMI	55 (43.31)	72 (56.69)	1.00	—	—	—
	URRBMI	224 (60.87)	144 (39.13)	2.04 (1.35 ~ 3.06)	<0.01	2.07 (1.37 ~ 3.12)	<0.01
Involved in the targeted disease reimbursement program ^c^	Yes	73 (47.71)	80 (52.29)	1.00	—	—	—
	No	207 (60.88)	133 (39.12)	1.71 (1.16 ~ 2.51)	0.01	NA	NA

^a^ Data was missing for five patients

^b^ Data was missing for one patients

^c^ Data was missing for three patients

^d^ Adjusted for study sites, age, sex, education, type of residence, level of income, type of health insurance, status of enrollment in the targeted disease reimbursement program and years of living with Type 2 DM. Data without statistically significant were not shown

## Discussion

In our study, more than half of the participants did not obtain services recommended the regional practice guideline of DM case management developed for the patients with Type 2 DM. We found one-third of the patients had less than four follow-up visits in a year. Around half of patients neither had recommended annual blood lipid test or screening for nephropathy and eyes and the proportion of annual HbA1c test was the lowest. Type of health insurance coverage was significantly associated with accessing recommended care after adjusting for participants’ demographic and other socio-economic status.

In China, universal coverage of essential public health services is one of the priorities of the Chinese health system reform launched in 2009 [6]. The essential public health service package had nine components in 2009, which included health management for patients with hypertension and diabetes [12]. One study conducted in the three provinces located in the eastern, central and western China respectively reported that 90% of people with hypertension or diabetes had at least one follow-up visit, while around half of them had regular follow-up visits and only one-third of the patients had blood glucose controlled in normal range [12], which was consistent with our findings regarding the frequency of follow-up visits. Our study added the evidence that some recommended tests for Type 2 DM management were not properly taken. The proportion of having HbA1c was the lowest, less than 10%. According to a prospective observational study in UK, any reduction in HbA1c was significantly associated with the reduction in risks of diabetes related clinical complications among Type 2 DM patients [13]. However, sub-standard management for people with Type 2 DM may jeopardize efforts to monitoring disease progression and preventing from complications and intensive care.

The previous studies in China and other countries found that age, sex and socio-economic status of patients with Type 2 DM and severity of disease were associated with the frequency of visiting doctors and the use of services [14–18]. In our study, the participants living with Type 2 DM more than five years were more likely to have regular follow-up visits. This may indicates that they have better awareness or demands for monitoring and controlling disease progression. In our study, another common factor associated with the frequency of visits and having various recommended tests was type of health insurance.

We found that the patients covered by UEBMI were more likely to have a standard management with regular visits and having recommended tests than those with coverage of URRBMI. Likewise, other previous studies in China found that people covered by UEBMI often used more and more expensive services than people covered by URRBMI and NCMS [19–21]. In general, UEBMI had a comprehensive coverage and provided higher reimbursement for outpatient services than the urban and rural residence health insurance schemes [22]. Despite a targeted disease reimbursement program was introduced in URRBMI to provide up to US$ 160 for outpatient reimbursement annually that was 10 times outpatient reimbursement for non-targeted diseases [5], only one-third participants were enrolled in the targeted disease reimbursement program. A possible explanation is that the patients with Type 2 DM either were not informed of the targeted disease reimbursement program or nor they know how to enrol in the program. In addition, financial difficulty was cited as a common reason for not having DM care in our study published elsewhere [5]. Without appropriately preventive interventions in the early stage of the disease, the disease progression may require more and more expensive care, which will cause heavy burden for both households with people living with DM and health system.

This study has several limitations. The participants of this study were identified from the register of non-communicable chronic diseases in local primary health centers. We failed to include patients with Type 2 DM who were not registered. These people may be more disadvantaged on having recommended DM management. Regarding the frequency of follow-up visit, DM patients with severe complications were recommended to have a follow-up visit every month. We were not able to identify disease status of the participants due to unable to access to their medical records. Thus, the recommended minimum four follow-up visits in a year for all patients with Type 2 DM were used as a standard measure in this study. In addition, this study only included two areas (out of total 38 district/counties) of Chongqing and only one township and one community in each study site were selected which yielded a relatively small sample size. This study can be viewed as a case study and generalizations to other areas should be made with cautions.

## Conclusion

Case management for patients with Type 2 DM was not effectively implemented in terms of frequency of follow-up visits and recommended tests over one-year period, as indicated in the regional practice guideline. Our study offers evidence that suggests actions for improving health system performance can be taken to better manage DM patients in China. Firstly, community and facility-based health education will be essential to raise patients’ awareness of the importance of Type 2 DM management. Furthermore, the outpatient service benefit package of health insurance schemes, especially for rural and urban unemployed residences should be increased in order to encourage the use of services for chronic diseases management. Such investment will be cost effective when more and intensive DM related cares would be avoided through effective case management. Moreover, this financial protection strategy should be widely disseminated and be beneficiary-friendly, given most patients with chronic diseases were elderly. In addition, it needs further study to understand perceptions of health managers and healthcare providers on chronic cases management in order to develop relevant strategies (e.g. pay-for-performance payment, in-services training, field support and supervision etc.) to improve adherence to evidence-based practices and health outcomes.

## Abbreviations

95% CI: 95% confidence intervals
DJ: A county of Chongqing, China
DM: Diabetes mellitus
GF: A township of Chongqing, China
GX: A community of Chongqing, China
HbA1c: Hemoglobin A1C
LX: A township of Chongqing, China
NCDs: Non-communicable diseases
NCMS: New Rural Cooperative Medical Scheme
OR: Odds Ratios
SFQ: A community of Chongqing, China
UEBMI: Urban Employed Basic Medical Insurance
URBMI: Urban Residence Basic Medical Insurance
URRBMI: Urban–rural Residence Basic Medical Insurance
YB: A district of Chongqing, China
